# Supplementary material for: Visualizing orthogonal RNAs simultaneously in live mammalian cells by fluorescence lifetime imaging microscopy (FLIM)
Source: Nat Commun. 2023 Feb 16;14:867. doi: 10.1038/s41467-023-36531-y (PMC9935525; doi:10.1038/s41467-023-36531-y)
Supplement: Supplementary file 1 — Supplementary Information [file 41467_2023_36531_MOESM1_ESM.pdf]

Supplementary Information

Visualizing orthogonal RNAs simultaneously in live mammalian cells by fluorescence lifetime imaging microscopy (FLIM)

Nadia Sarfraz[1], Emilia Moscoso[1], Therese Oertel[1], Harrison J. Lee[1], Suman Ranjit[2, 3], Esther Braselmann[1]*

1 Department of Chemistry, Georgetown University, Washington, District of Columbia, USA
2 Department of Biochemistry and Molecular & Cellular Biology, Georgetown University, Washington, District of Columbia, USA
3 Microscopy & Imaging Shared Resource, Georgetown University, Washington, District of Columbia, USA

*corresponding author

Supplementary Figures

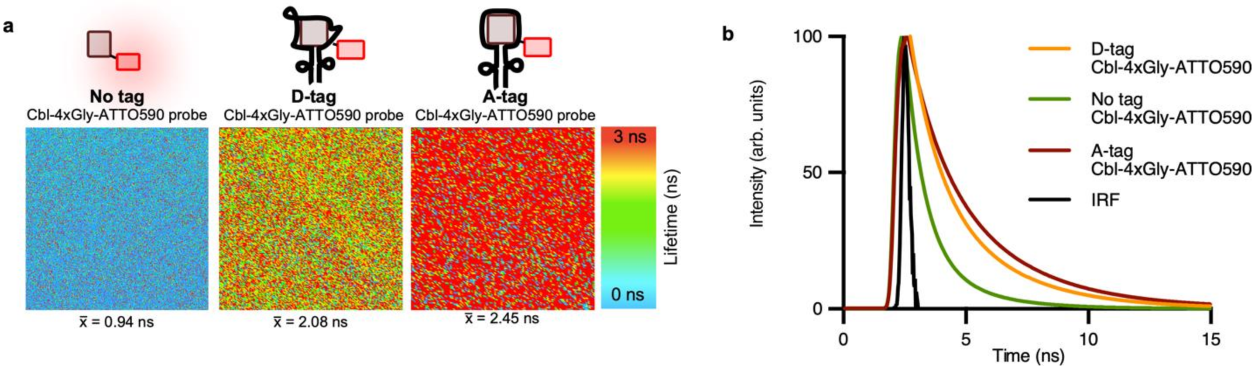

c

	Literature Avg. Intensity Lifetime Braselmann et al. (2018)	Obtained Avg. Intensity Lifetime	Obtained Avg. Amplitude Lifetime
**Cbl-4xGly-ATTO590 No tag**	1.44 ns	1.32 ns	1.03 ns
		1.24 ns	0.96 ns
		1.19 ns	0.92 ns
		1.12 ns	0.85 ns
		x̄ = 1.22 ns	x̄ = 0.94 ns
**Cbl-4xGly-ATTO590 D-tag**	2.42 ns	2.61 ns	2.20 ns
		2.58 ns	2.09 ns
		2.50 ns	2.05 ns
		2.43 ns	1.96 ns
		x̄ = 2.53 ns	x̄ = 2.08 ns
**Cbl-4xGly-ATTO590 A-tag**	2.98 ns	2.92 ns	2.54 ns
		2.98 ns	2.63 ns
		2.80 ns	2.40 ns
		2.67 ns	2.24 ns
		x̄ = 2.84 ns	x̄ = 2.45 ns

Supplementary Figure 1. *In vitro* fluorescence lifetime of Cbl-4xGly-ATTO590 in the presence and absence of Riboglow RNA (A tag or D tag). (a) Fluorescence lifetime of samples was measured in drops on a microscope. False color scale illustrates mean average lifetimes across multiple repeat measurements (n=4). (b) Representative decay curves of sample shown in (a), plotted with the Instrument Response Function (IRF). (c) Summary table of previously reported intensity lifetime measurements and obtained intensity and amplitude weighted fluorescence lifetimes.[1]

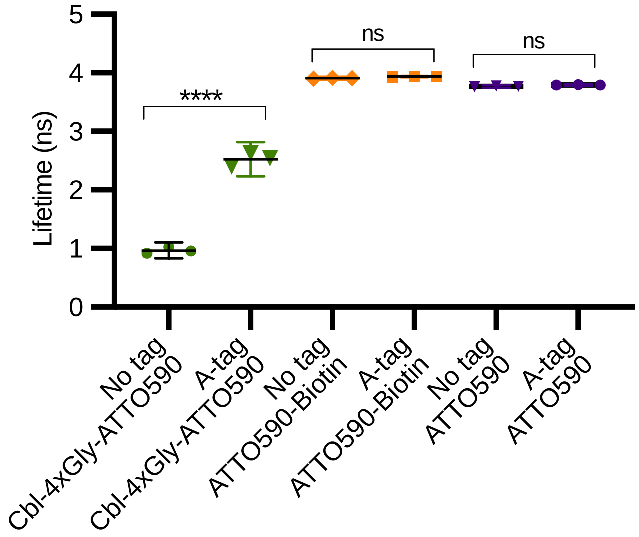

Supplementary Figure 2. Average *in vitro* fluorescence lifetime of ATTO590 probe variants in the presence and absence of purified Riboglow RNA A tag. ATTO590 variants used were Cbl-4xGly-ATTO590, ATTO590-Biotin and free ATTO590.

Mean average lifetimes for n=18 independent samples examined over 3 independent experiments (1 symbol = 1 measurement, ns: p≤ 0.5; ****:p≤ 0.0001). Error bars indicate mean and standard deviation (+/-SD). One-way ANOVA (95% confidence limit); post hoc test (Tukey HSD).

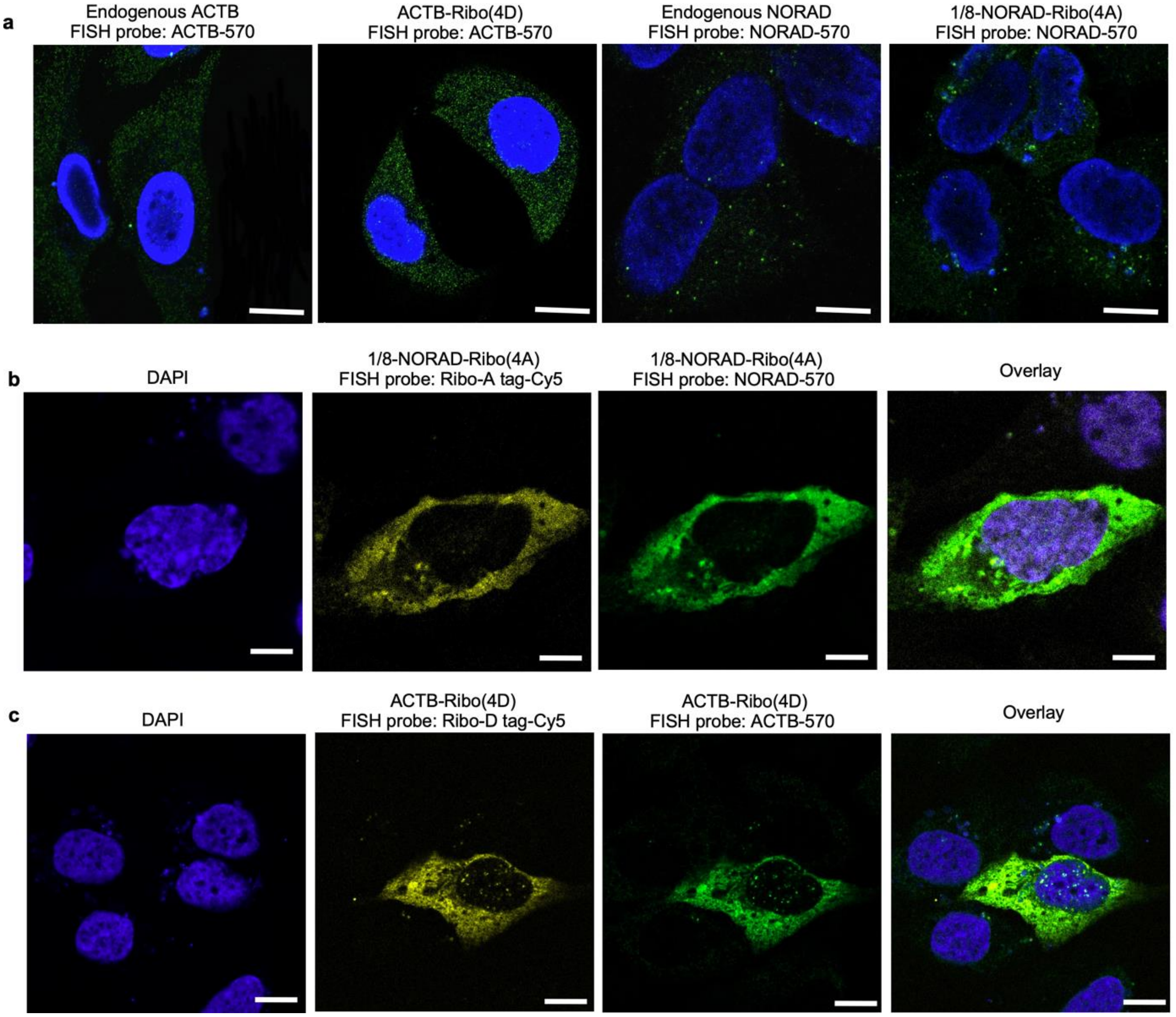

Supplementary Figure 3. Localization of endogenous RNAs in U-2 OS cells vs. U-2 OS cells transfected with Riboglow-tagged constructs by fluorescence in situ hybridization (FISH). (a) U-2 OS cells were fixed. FISH probes targeting the ACTB sequence were used to probe endogenous ACTB mRNA vs. U-2 OS cells transfected with ACTB-Ribo(4D). U-2 OS cells were fixed and FISH probes targeting the NORAD sequence were used to probe endogenous NORAD vs. 1/8-NORAD produced from a plasmid. (b) U-2 OS cells were transfected with 1/8 NORAD-Ribo(4A) and fixed with FISH probes targeting the NORAD sequence (green) and FISH probes targeting the Ribo-A tag (yellow). Nucleus stained with DAPI. (c) U-2 OS cells were transfected with ACTB-Ribo(4D) and fixed with FISH probes targeting the ACTB sequence (green) and FISH probes targeting the Ribo-D tag (yellow). (4 repetitions, 300 cells, blue = DAPI stained nucleus). Scale bar = 10 µm.

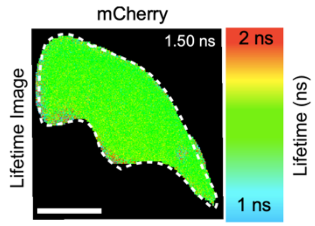

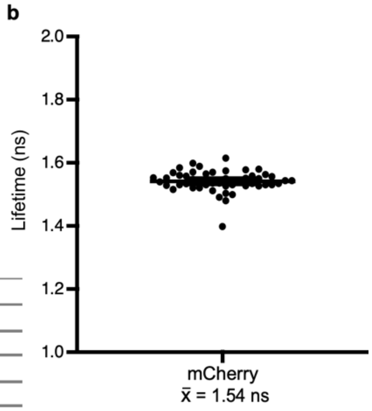

c

Literature τ of mCherry	τ (ns)
Nakahata et al.	1.54 ns
Poland et al.	1.4 ns
Seefeldt et al.	1.46 ns
Itoh et al.	1.42 ns
This study	1.54 ns

Supplementary Figure 4. Fluorescence lifetime imaging microscopy (FLIM) of live U-2 OS cells transfected with a plasmid producing mCherry. (a) Visual representation of average fluorescence lifetime with a false color scale. For each cell, the average lifetime and intensity for a region of interest that contains the entire cell was measured (dotted line). The resulting average lifetime value as defined in the methods for the representative cell is shown (1.50 ns). Scale bar = 10 µm. (b) Lifetimes of 50 cells (3 independent experiments) transfected with a plasmid producing mCherry (• =1 cell), mean average across all cells ($\bar{x}$) listed. (c) Literature values for fluorescence lifetime (τ) of mCherry in live mammalian cells compared to this study.[2–5] Error bars indicates mean and standard deviation (+/-SD).

a

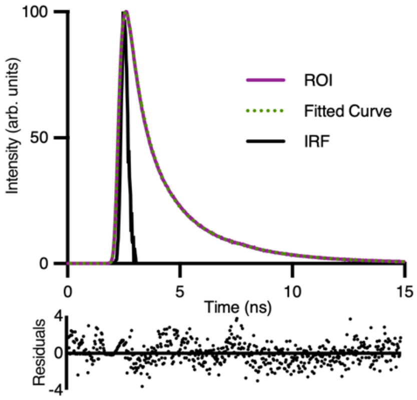

Untransfected-590	
**Parameters**	
**A1 (arb. units)**	16.0 ±0.13
**A2 (arb. units)**	24.0 ± 0.38
**τ_1 (ns)**	3.279 ± 0.0077
**τ_2 (ns)**	1.240 ± 0.011
**τ_3 (ns)**	0.4521 ±0.0062
**Shift IRF (ns)**	0.00900 ±0.00033
**Bkgr IRF (arb. units)**	3.7 ± 7.1
**I_1 (arb. units)**	2074 ± 9.3
**I_2 (arb. units)**	1169 ± 12
**I_3 (arb. units)**	519 ± 12
**I_{sum} (arb. units)**	3763 ± 4.1
**A_{sum} (arb. units)**	68.100 ± 0.072
**Avg τ_{int} (ns)**	2.2550 ± 0.0013
**Avg τ_{amp} (ns)**	1.3810±0.0022
**A rel 1**	0.2322 ±0.0025
**A rel 2**	0.3461 ±0.0052
**A rel 3**	0.4217 ±0.0044

b

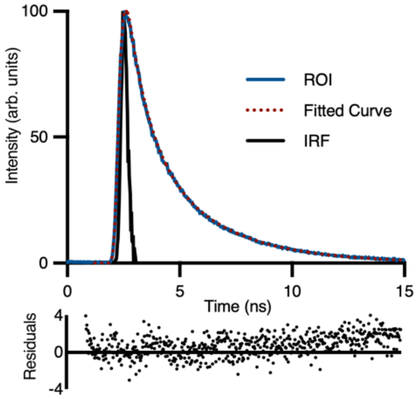

ACTB-Ribo(4A)590	
**Parameters**	
**A1 (arb. units)**	5.990 ±0.054
**A2 (arb. units)**	7.470 ± 0.068
**τ_1 (ns)**	3.190 ± 0.017
**τ_2 (ns)**	0.954 ±0.018
**Shift IRF (ns)**	0.00019 ±0.00077
**Bkgr IRF (arb. units)**	68 ± 25
**I_1 (arb. units)**	764 ± 3.7
**I_2 (arb. units)**	285 ± 4.8
**I_{sum} (arb. units)**	1049.0 ± 1.5
**A_{sum} (arb. units)**	13.00 ± 0.0.04
**Avg τ_{int} (ns)**	2.5830 ± 0.0046
**Avg τ_{amp} (ns)**	1.950±0.0011
**A rel 1**	0.4451 ±0.0045
**A rel 2**	0.5549 ± 0.0045

c

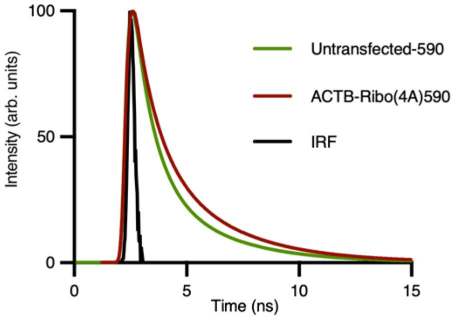

d

**Symbol**	A	τ_{amp}
**Definition**	Amount of each fluorescing component	Average amplitude fluorescence lifetime
**n**	3	2
**Equation**	$A_{rel} = \frac{A_i}{\sum_{i=1}^{n} A_i}$	$\tau_{amp} = \sum_{i=1}^{n} A_{rel} \cdot \tau_i$
**Untransfected-590**	$A_{rel\,1}$ =0.2322 ±0.0025<br>$A_{rel\,2}$ =0.3461 ±0.0052<br>$A_{rel\,3}$ =0.4217 ±0.0044	τ_{amp} = 1.3810±0.0022
**ACTB-Ribo(4A)590**	$A_{rel\,1}$ =0.4451 ±0.0045<br>$A_{rel\,2}$ =0.5549 ± 0.0045	τ_{amp} =1.9500±0.0011

Supplementary Figure 5. Workflow for fitting and analysis of fluorescence lifetime imaging microscopy (FLIM) of live mammalian cells with indicated reporters. (a, b) Average fluorescence for representative live mammalian cells with listed reporter. A region of interest that represents the whole cell was defined for each representative cell and fit using a multiexponential reconvolution fit. The resulting data and decay curves for the whole cells (with residual maps) are shown. (c) Overlay of decay curves from (a, b). (d) Overview of fitting parameters, where A is defined as the amount of fluorescing components and τ_{amp} is the average lifetime. In the FLIM analysis workflow throughout this study, the listed equations were used to obtain a final τ_{amp} for each cell.

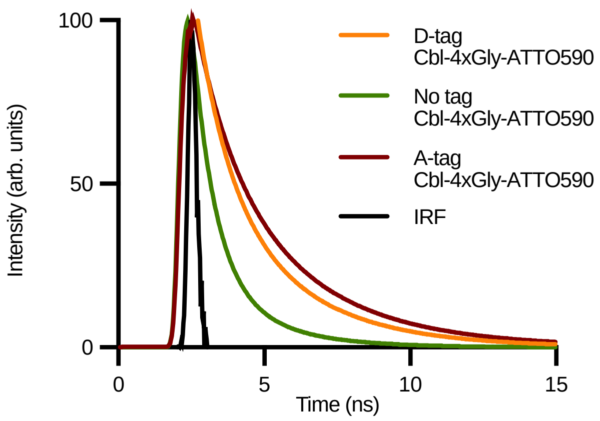

Supplementary Figure 6. Representative fluorescence lifetime decay curves of whole cells producing various Riboglow variants, plotted with the Instrument Response Function (IRF). The lifetime decay was determined for a region of interest that includes the entire cell. Representative cells that produced the lifetime values shown here are presented as cell images in Figure 2, panel b and as a data point in Figure 4, panel a.

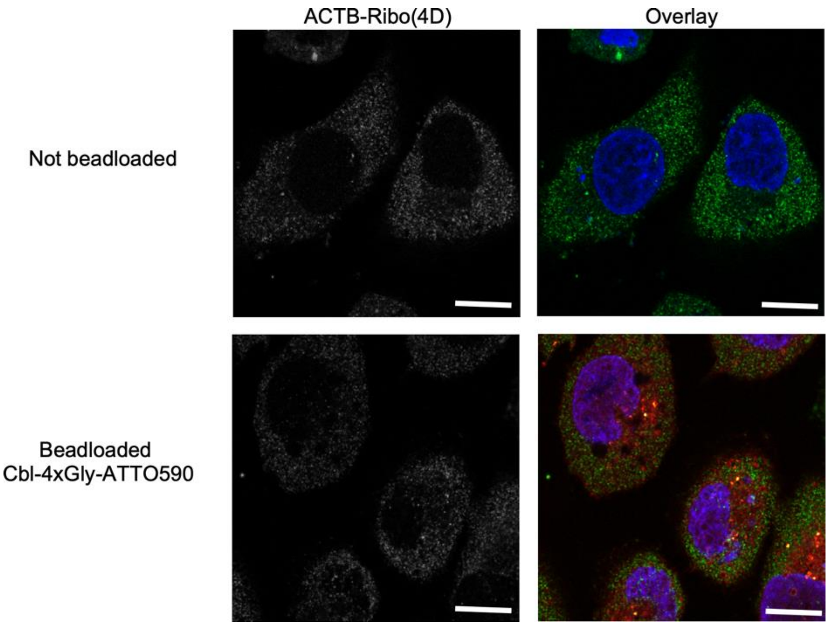

Supplementary Figure 7. Representative FISH images comparing ACTB mRNA localization patterns with and without bead loading. U-2 OS cells were fixed and FISH probes targeting the ACTB sequence used. Probe Cbl-4xGly-ATTO 590 was bead loaded. Scale bar = 10 µm. Cells were transfected with ACTB-Ribo(4D) and bead loaded with Cbl-4xGly-ATTO590 probe (n = 21 cells over two independent experiments).

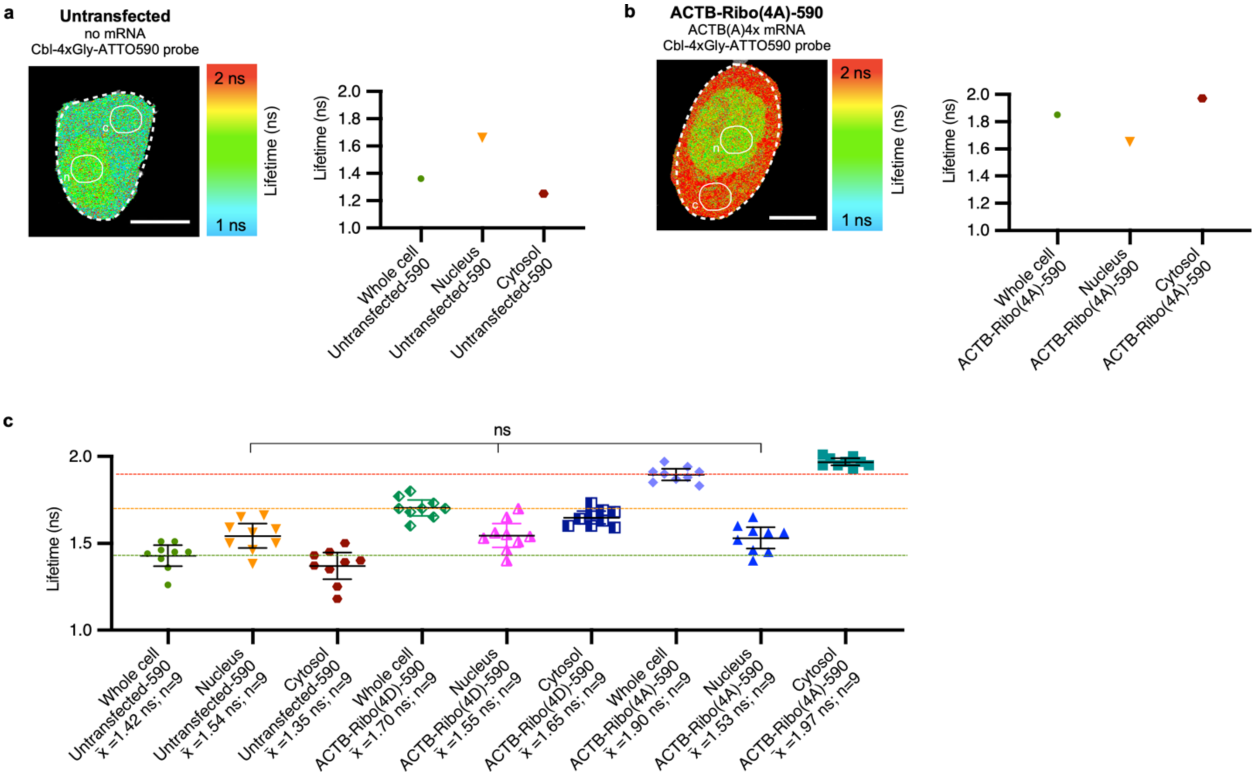

Supplementary Figure 8. Comparison of fluorescence lifetime between U-2 OS cytosol and nuclear signals. (a, b) Representative images and measurements of lifetime values in regions of an untransfected cell vs. a cell transfected with a plasmid to produce ACTB-Ribo(A)4x mRNA, both loaded with Cbl-4xGly-ATTO590 probe. Cells in (a) and (b) are part of the dataset in (c). Scale bar = 10 µm. Comparison of average fluorescence lifetime between U-2 OS whole cell, cytosol and nuclear signal across cell populations (a, b). Mean average lifetimes and intensities for 5 independent experiments per condition, 27 cells (1 symbol = 1 measurement, ns: $p \leq 0.5$; *: $p \leq 0.05$; **: $p \leq 0.01$; ***:$p \leq 0.001$; ****:$p \leq 0.0001$). Dotted lines represent the mean of the lifetime for benchmarks established from ACTB tagged mRNA in Fig. 3. Error bars indicate mean and standard deviation (+/- SD).One-way ANOVA (95% confidence limit); post hoc test (Tukey HSD), ns = non-significant.

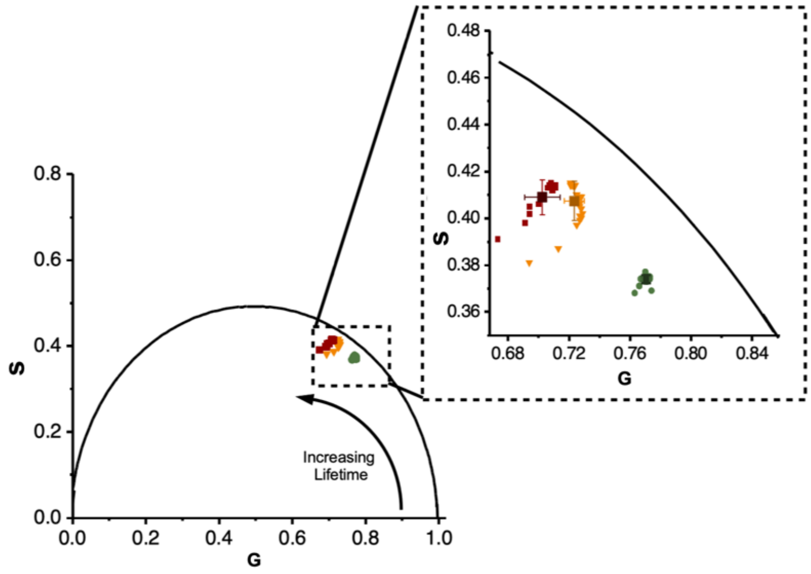

Supplementary Figure 9. FLIM time correlated single photon counting (TCSPC) imaging of live mammalian cells fitted with phasor analysis. (a) Graphical representation of lifetime coordinates **G** and **S** shown on a phasor plot, with direction of increasing lifetime depicted by an arrow. Green: untransfected cells, orange triangle: transfected cells producing ACTB-Ribo(4D)-590, red square: transfected cells producing ACTB-Ribo(4A)-590. One dot represents one live cell. Error bars indicate mean and standard deviation (+/-SD). Mean average lifetimes for 4 independent experiments per condition, n = 65 cells.

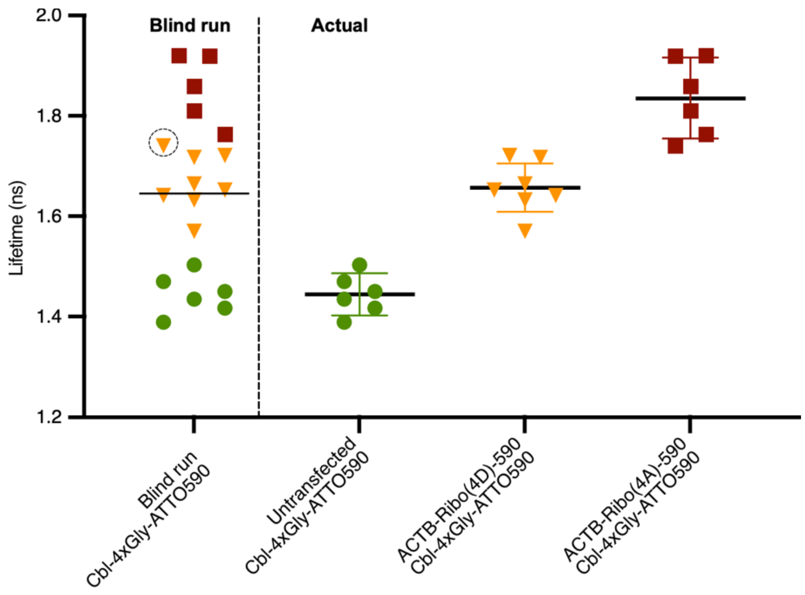

Supplementary Figure 10. Comparison of results from a blind run to evaluate robustness of data extraction and method for our Riboglow-FLIM system. Samples were imaged by FLIM, labeled in a non-descriptive fashion and multiexponential reconvolution fitting was done as outlined in Methods / Supplementary Note 1. Cells were then categorized as untransfected (green circle), transfected to produce ACTB-Ribo(4A)-590 (red square) or ACTB-Ribo(4D)-590 (orange triangle). The data to the left of the dotted line are the complete results of the blind run, while the bars to the right of the dotted line show the known categories of the cells after unblinding. One cell was misidentified and is shown circled in the plot. Mean average lifetimes for 6 independent experiments per condition, n=19 cells. Error bars indicate mean and standard deviation (+/-SD).

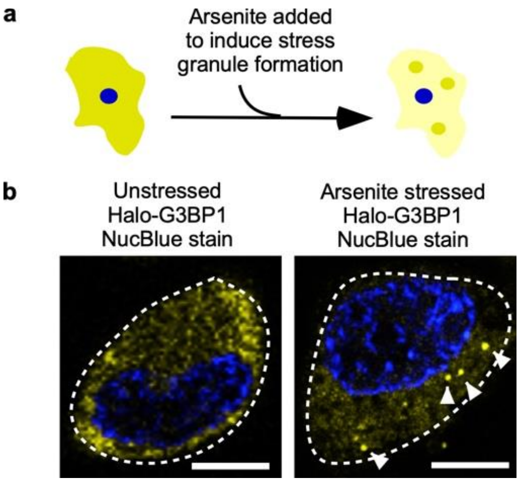

Supplementary Figure 11. Model system for RNA recruitment to mammalian stress granules. (a) Cartoon representation of stress granule formation upon arsenite stress. (b) Halo-G3BP1 cells stained with Janelia Fluor 646 dye were imaged in the presence and absence of arenite stress and show the formation of stress granules, identified by the marker protein G3BP1 (white arrow). Nucleus = blue. Representative cell from 3 experiments, 19 cells, scale bar = 10 µm.

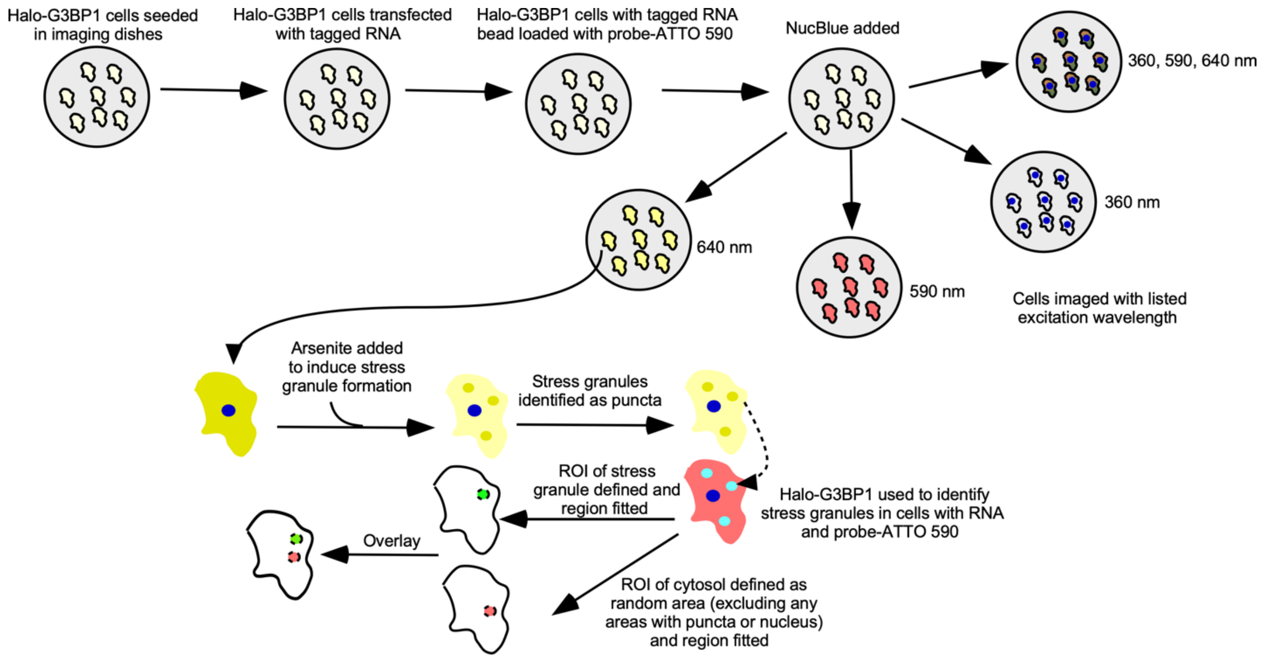

Supplementary Figure 12. Workflow to identify stress granules and quantify Riboglow lifetime values in stress granules. Stress granules were identified using a fluorescent marker protein, Halo-G3BP1, which has been shown to localize in stress granules in mammalian cells (Supplementary Figure 11).[1] Regions of interest of both cytosol and stress granules were selected through inspection of Halo-G3BP1 granule formation. Cells were imaged with different excitation wavelengths as follows: 360 nm excitation to identify nuclei (NucBlue), 590 nm excitation to identify cells that were loaded with the ATTO590-containing Riboglow probe and 640 nm excitation wavelength to identify cells where stress granules were formed. An overlay of these channels was collected and the fluorescence lifetime for Riboglow was measured. Overlay of all channels allowed assigning fluorescence lifetime region of interest to stress granules and random cytosol regions.

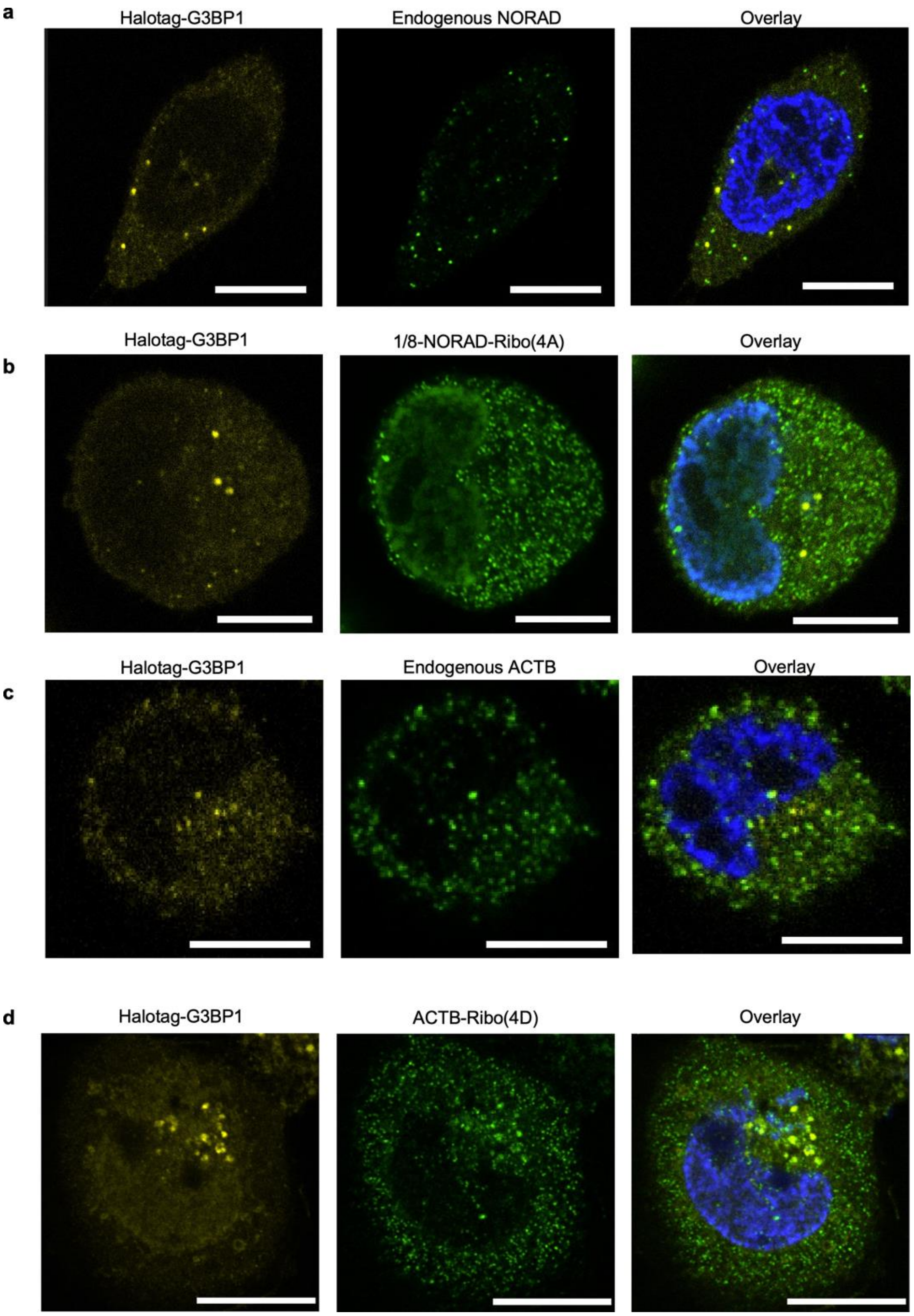

Supplementary Figure 13. Comparison of endogenous RNA and Riboglow-tagged reporter RNA produced after transient transfection during stress. U-2 OS cells producing Halo-G3BP1 were stressed with arsenite and Halo-G3BP1 was stained with JF646 (yellow, left column). The indicated RNA was stained by fluorescence in situ hybridization (FISH) (green, middle column). FISH probes targeting the ACTB and NORAD sequence were used. Right column: overlay, blue nucleus added. (a) endogenous NORAD, (b) transfected 1/8-NORAD-Ribo(4A), (c) endogenous ACTB mRNA, (d) transfected ACTB-Ribo(4D). Scale bar = 10 µm. (2 repetitions, 233 cells).

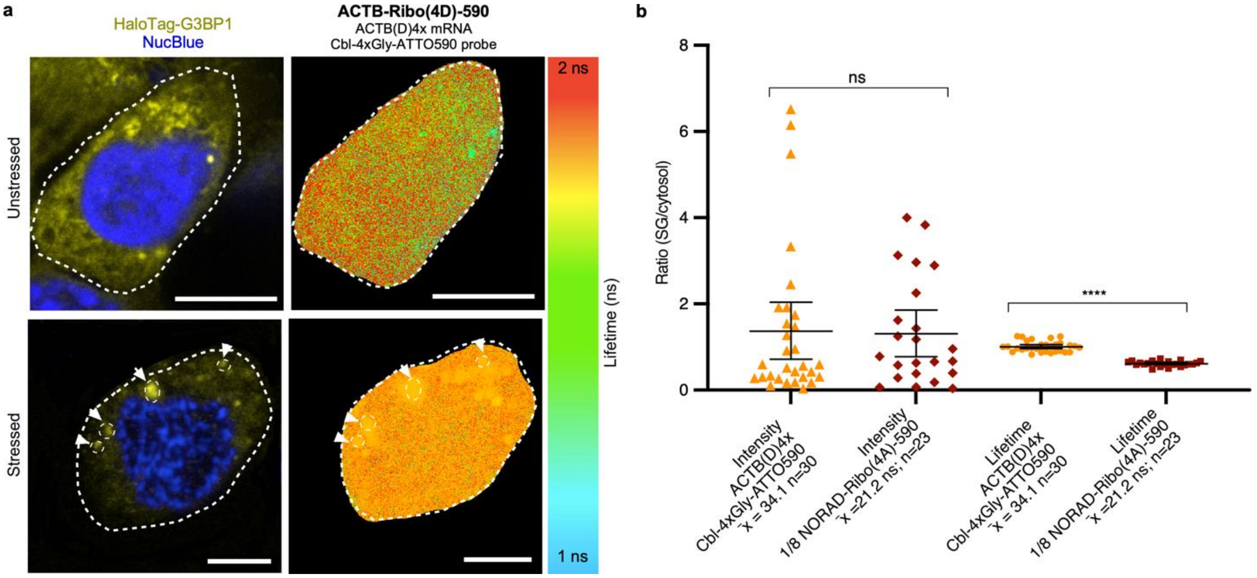

Supplementary Figure 14. (a) ACTB-Ribo(4D)-590 mRNA localization in unstressed and arsenite-stressed conditions. U-2 OS cells that produce Halo-G3BP1 endogenously were stained with the JF646 dye and imaged in the presence and absence of arsenite stress. Stress granules were identified via the JF646 dye in arsenite stress conditions (white arrow). Nucleus = blue. Representative cell from 4 experiments, 53 cells, scale bar = 10 µm. (b) Fluorescence intensity and average fluorescence lifetime quantification ratios (SG/cytosol) in stress granules (SG). 4 independent experiments per condition, 53 cells (1 symbol = 1 measurement, ns: $p \leq 0.5$; *: $p \leq 0.05$; **: $p \leq 0.01$; ***:$p \leq 0.001$; ****:$p \leq 0.0001$). Error bars indicate mean and standard deviation (+/-SD).One-way ANOVA (95% confidence limit); post hoc test (Tukey HSD).

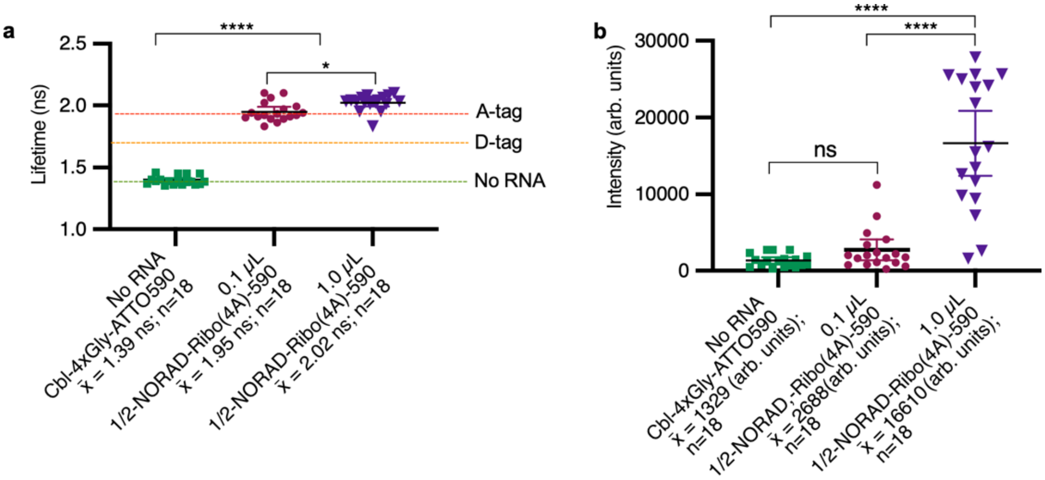

Supplementary Figure 15. Effect of varying production of RNA reporter levels in cells on fluorescence lifetime of Riboglow (a) and fluorescence intensity values (b). U-2 OS cells were transfected with varying amounts of plasmid DNA to produce RNA reporter 1/2-NORAD-Ribo(4A)-590 and loaded with Cbl-4xGly-ATTO590 (1 symbol = 1 cell). Fluorescence lifetime and intensity values for regions of interest (ROI) that include whole cells were extracted. Intensity values per cell were obtained and fluorescence lifetime was calculated by iterative reconvolution and the average value for the entire ROI is reported (as outlined in Methods). Mean average lifetimes for 3 independent experiments per condition, 54 cells (1 symbol = 1 measurement, ns: $p \leq 0.5$; *: $p \leq 0.05$; **: $p \leq 0.01$; ***:$p \leq 0.001$; ****:$p \leq 0.0001$). Dotted lines (a) represent the mean of the lifetime for benchmarks established from ACTB tagged mRNA in Fig. 3. Error bars indicate mean and standard deviation (+/-SD). One-way ANOVA (95% confidence limit); post hoc test (Tukey HSD).

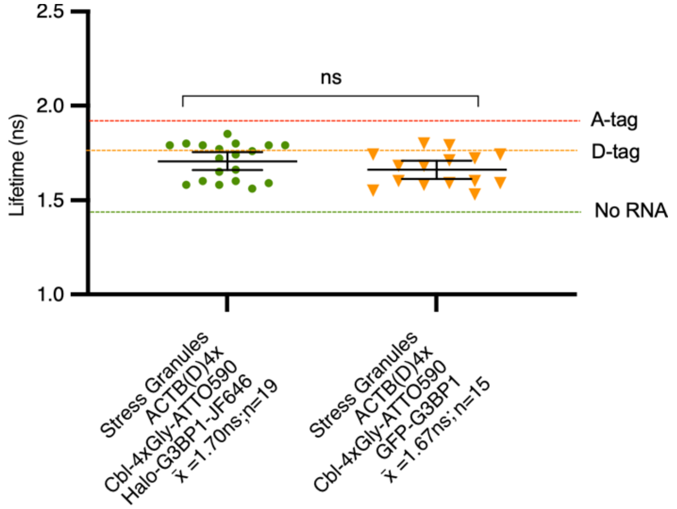

Supplementary Figure 16. Fluorescence lifetime of ACTB mRNA tagged with Riboglow (ACTB-Ribo(4A)-590) after recruitment to stress granules in two different cell lines. The RNA reporter was transfected in two different U-2 OS cell lines producing different versions of the stress granules marker protein G3BP1. Left: cells produced endogenous G3BP1 tagged with a HaloTag that binds the dye JF646; right: cells were co-transfected with a plasmid to produce GFP-G3BP1 (34 cells, 4 experiments). Dotted lines represent the mean of the lifetime for benchmarks established from ACTB tagged mRNA in Fig. 3. Error bars indicate mean and standard deviation (+/-SD). One-way ANOVA (95% confidence limit); post hoc test (Tukey HSD), ns: p≤ 0.5.

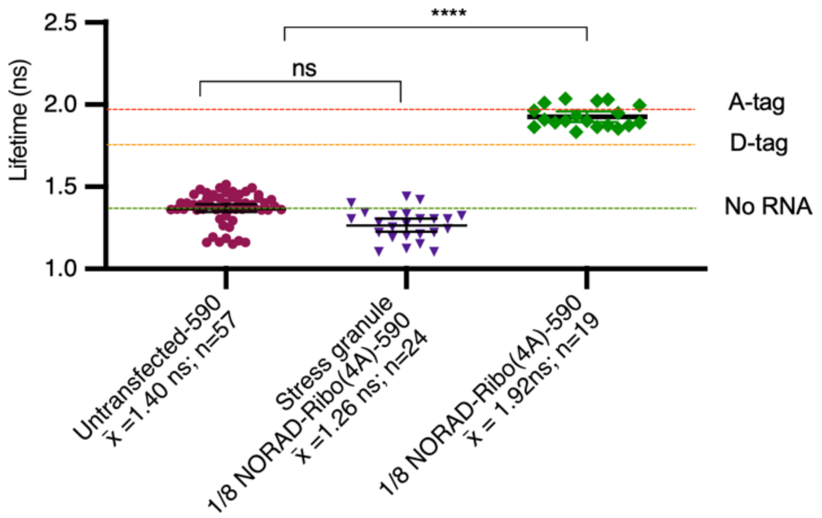

Supplementary Figure 17. Comparison of fluorescence lifetime in stress granules where the Riboglow-tagged RNA was excluded for cells producing the 1/8 NORAD-Ribo(4A)-590 reporter (purple triangles) *versus* unstressed cells where no tagged RNA was present (untransfected, pink circles). The lifetime values of the whole cells producing 1/8 NORAD-Ribo(4A)-590 are shown in comparison (green symbols). The Cbl-4xGly-ATTO590 probe was present in all cases. Mean average lifetimes for 4 independent experiments per condition, 100 cells (1 symbol = 1 measurement, ns: $p \leq 0.5$; *: $p \leq 0.05$; **: $p \leq 0.01$; ***:$p \leq 0.001$; ****:$p \leq 0.0001$). Dotted lines represent the mean of the lifetime for benchmarks established from ACTB tagged mRNA in Fig. 3. Error bars indicate mean and standard deviation (+/-SD). One-way ANOVA (95% confidence limit); post hoc test (Tukey HSD).

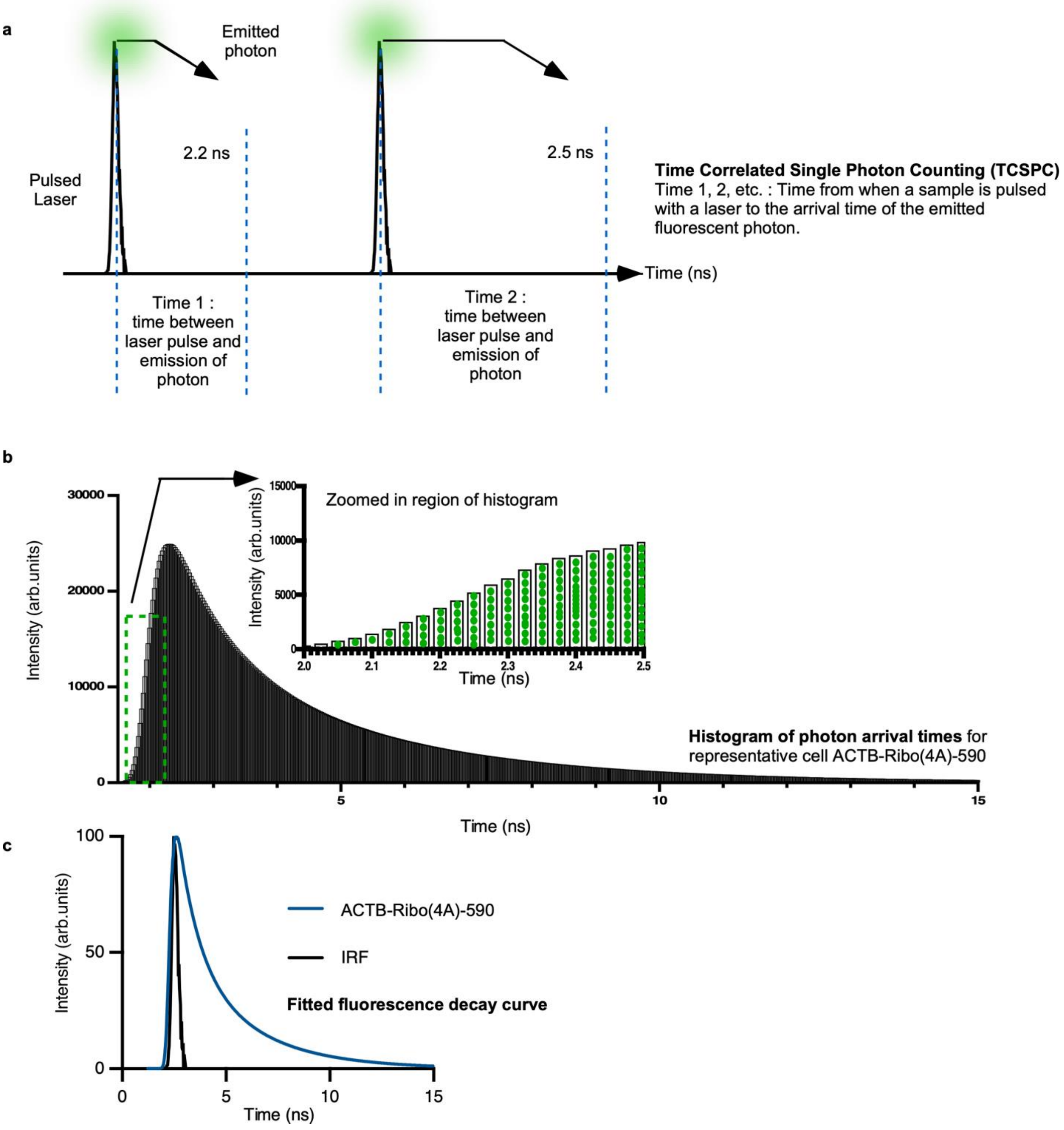

Supplementary Figure 18. Fluorescence lifetime imaging microscopy using time correlated single photon counting (FLIM-TCSPC) system and measurements for live mammalian cells with a defined region of interest showing (a) the time of laser pulse to photon arrival time (indicated to left of blue line) at each pixel summarized as a histogram of arrival times for a region of interest in (b), yielding a final decay curve that can be normalized and fitted (c). Representative cell from 12 experiments in which n=48 cells under reported condition.

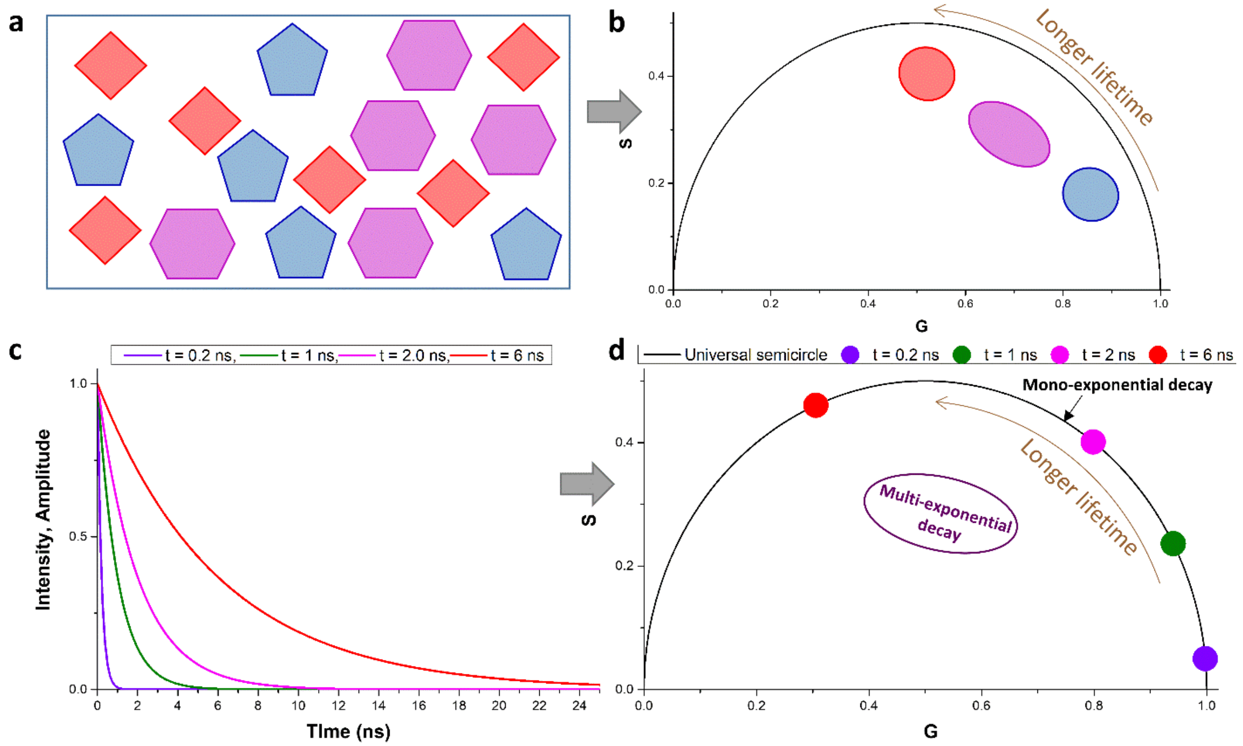

Supplementary Figure 19. Concept of the phasor approach. (a) Cartoon representation of cells in a field of view of the microscope. There are three types of cells in this field of view with three different fluorescence lifetime signatures. The red squares have a dye with the longest fluorescence lifetime and blue pentagons represent a dye with the shortest lifetimes. The pink hexagons contain both red and blue dyes and have an average lifetime that is in between the individual lifetimes. (b) Phasor representation of the fluorescence decays from the sample in (a). After phasor transformation, shorter lifetimes appear at a smaller phase angle and the longer lifetimes appear at a larger phase angle, as shown in the figure. The position of the phasor positions calculated from the decays are represented using the same color code. As the hexagons contain both blue and red dyes, their positions are along the line joining the phasor positions of squares and pentagons. (c) Calculated fluorescence intensity decays based on 4 examples of mono-exponential lifetimes – 0.2 ns (violet), 1.0 ns (green), 2.0 ns (pink) and 6.0 ns (red). (d) After phasor transformation, the mono-exponential decays appear at the universal semi-circle (black). The corresponding positions of the decays in (c) are shown after phasor transformation for a phasor calculated for 40 MHz laser frequency. The image pixels where the decays are multi-exponentials appear inside the semicircle.

Supplementary Tables

Supplementary Table 1

Currently available genetically encoded fluorophore-based RNA tagging and tracking systems for use in live mammalian cells.

Name	Description	RNA Tag Size	Feasibility of Color-Multiplexing
**RhoBAST[6]**	Fluorescence light-up RNA aptamer (FLAP) binds rhodamine	16 copies of RhoBAST sequence for mRNA imaging in mammalian cells (16x 55 nt = 825 nt)[6]	Requires color-orthogonal system, concept demonstrated[7]
**Spinach[8]**<br>**Broccoli[9]**	Evolved fluorogenic RNA aptamers bind DFHBI and derivatives	Latest Broccoli variant[10] uses 6 repeats of F30-2xdBroccoli (6x 234 nt = 1404 nt)	Requires color-orthogonal systems, Broccoli-BI and Squash-DFHO combined in ratiometric sensor[11]
**MS2 stem loop (SL) repeats[12]**	Phage MS2 SL binds MS2 coat protein tagged with fluorescent protein	24 copies of MS2 SL for mRNA imaging (24x 61 nt = 1354 nt)	Requires orthogonal SL repeat / binding protein pair with second fluorescent protein, i.e. PP7[13]
**Mango[14]**	Evolved fluorogenic RNA aptamer binds thiazole derivatives	1 copy sufficient when in U6 promoter context (25 nt for core Mango IV sequence)[15]	Requires color-orthogonal system, multiplexing of Mango and Peach demonstrated *in vitro*[16]
**Peppers[17]**	Evolved RNAs that induce turn-on fluorescence upon binding probe	43 nt for 1 copy[17]	Requires color-orthogonal system, not demonstrated
**o-Choral / Gemini-561[18]**	Fluorogenic dimer of self-quenched sulforhodamine B dye binds corresponding evolved dimerized aptamer	150 nt for 1 copy of o-Choral[18]	Requires color-orthogonal system, not demonstrated
**SRB[19] / DNB[20]**	DNB: Orthogonal RNA aptamers that bind dinitroaniline and sulforhodamine B, evaluated in bacteria, not in mammalian cells<br>SRB: RNA aptamer binds dyes with different colors	15 repeats of SRB-2: 920 nt[21]	SRB-2 and DNB multiplexing with color-orthogonal RNA aptamer platforms demonstrated[20,21]
**Riboglow[1,22]**<br>**(this study)**	RNA tag binds fluorescent probe, induces change in fluorescence intensity and lifetime	103 nt for 1xA tag[1] (this study)	Two RNA tag orthogonality demonstrated using lifetime (this study)

Supplementary Table 2

Summary of Riboglow feature properties (riboswitch-derived Cobalamin (Cbl) binding RNA tag, Cbl-fluorophore probe).

**Riboswitch-derived Cbl-binding RNA tag**	
**A (** 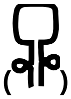 **)**	103 nt (1xA)<br>5′- GGC CUA AAA GCG UAG UGG GAA AGU GAC GUG AAA UUC GUC CAG AUU ACU UGA UAC GGU UAU ACU CCG AAU GCC ACC UAG GCC AUA CAA CGA GCA AGG AGA CUC -3′
**D (** 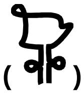 **)**	130 nt (1xD)<br>5′- GGU ACU GAA AGC GUG GUG GGA AAC AAU GUG AAA GUC AUU GAC UGU UCC UGC AAC GGU AAG CGC UUC GGC GCG AGU CCG AAU GCC ACC CAG UAA AGU CCG CUG UCG AGU GAA GGC CAG GAA AAG UCU AAC UCA -3′
**Cbl-fluorophore probe**	
**Cbl-4xGly-ATTO 590**<br>**(** 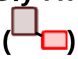 **)**	Excitation: 594 nm, Emission: 604 – 820 nm (Atto tech)
**Riboglow tag (RNA tag bound to probe)**[1]<br>(Braselmann et al., Nature Chem. Bio, 2018)	
**Ribo-(1A)-590**<br>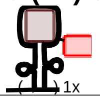	5.0x fluorescence increase *in vitro*
**Ribo-(1D)-590**<br>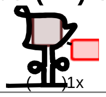	3.9x fluorescence increase *in vitro*

Supplementary Table 3

Comparison of commonly used fitting methods with representative data (TCSPC data from one cell per condition) showing robustness of data to ensure differentiation regardless of fitting procedure. τ_I (intensity-based lifetime) and τ_{amp} (amplitude-based lifetime).

	Tailfit			Reconvolution		
**Parameters (n)**<br>**Lifetime (τ)**	n=1<br>τ_{Amp} ; τ_I	n=2<br>τ_{Amp}	n=2<br>τ_I	n=1<br>τ_{Amp}; τ_I	n=2<br>τ_{Amp}	n=2<br>τ_I
**ACTB-Ribo(4A)-590**	2.52 ns	2.17 ns	2.72 ns	2.41 ns	1.90 ns	2.48 ns
**Untransfected-590**	2.09 ns	1.66 ns	2.28 ns	1.92 ns	1.44 ns	2.07 ns

Phasor Analysis:

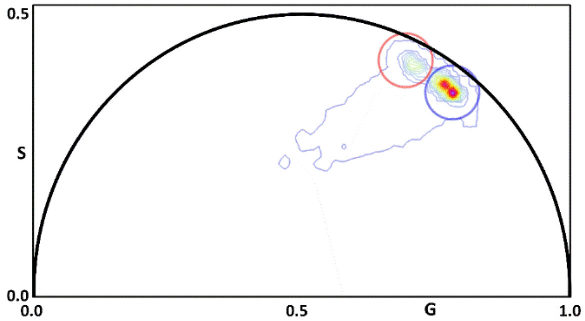

Sample name	Cursor selection	τ_{Phase} (ns)
**Cbl-4xGly-ATTO590**	Blue	1.86
**ACTB-Ribo(4A)-590**	Red	2.34

Supplementary Table 4

Statistical comparison of data listed in Figure 3.

Reported value	P-value pair	Mean	P-value (symbol)
**Intensity (arb. units)**	Untransfected-590: ACTB-Ribo(4D)-590	M1 = 2559.74<br>M2 = 7976.24	p = .00021 (***)
	Untransfected-590: ACTB-Ribo(1A)-590	M1 = 2559.74<br>M3 = 3271.00	p = .94414 (ns)
	Untransfected-590: ACTB-Ribo(4A)-590	M1 = 2559.74<br>M4 = 12601.17	p = .00000 (****)
	ACTB-Ribo(4D)-590: ACTB-Ribo(1A)-590	M2 = 7976.24<br>M3 = 3271.00	p = .00170 (**)
	ACTB-Ribo(4D)-590: ACTB-Ribo(4A)-590	M2 = 7976.24<br>M4 = 12601.17	p = .00213 (**)
	ACTB-Ribo(1A)-590: ACTB-Ribo(4A)-590	M3 = 3271.00<br>M4 = 12601.17	p = .00000 (****)
**Avg. lifetime (ns)**	Untransfected-590: ACTB-Ribo(4D)-590	M1 = 1.40<br>M2 = 1.70	p = .00000 (****)
	Untransfected-590: ACTB-Ribo(1A)-590	M1 = 1.40<br>M3 = 1.88	p = .00000 (****)
	Untransfected-590: ACTB-Ribo(4A)-590	M1 = 1.40<br>M4 = 1.89	p = .00000 (****)
	ACTB-Ribo(4D)-590: ACTB-Ribo(1A)-590	M2 = 1.70<br>M3 = 1.88	p = .00000 (****)
	ACTB-Ribo(4D)-590: ACTB-Ribo(4A)-590	M2 = 1.70<br>M4 = 1.89	p = .00000 (****)
	ACTB-Ribo(1A)-590: ACTB-Ribo(4A)-590	M3 = 1.88<br>M4 = 1.89	p = .98863 (ns)

Supplementary Note 1

Analysis and fitting of Riboglow-FLIM data

Fluorescence lifetime is an intrinsic characteristic property of a fluorophore that is exploited in Fluorescence Lifetime Imaging Microscopy (FLIM). The fluorescence lifetime of each pixel is detected by laser scanning microscopy using Time-Correlated Single Photon Counting (TCSPC). In TCSPC, the photon arrival times at the detector after pulsed laser excitation are recorded (Supplementary Figure 18).[23-24] We conducted TCSPC measurements for live mammalian cells transfected with plasmids producing RNA reporters and chemical probes listed in Supplementary Table 2. The photon arrival time at each pixel was recorded as a histogram of arrival times for a region of interest (ROI), defined as an individual cell (i.e., Figure 2) or a select cellular region (i.e., stress granules in Figure 5).

The acquired decay function at each pixel is then analyzed further to extract fluorescence lifetime values. We compared several possible fitting methods for live cells with just the Cbl-4xGly-ATTO 590 probe and live cells with the Cbl-4xGly-ATTO 590 probe that also produced RNA tagged with Riboglow RNA tag that binds to the probe, for example yielding the mRNA reporter ACTB-Ribo(4A)-590 (Supplementary Table 2). ACTB-Ribo(4A)-590 was used as a model to compare fitting workflows. We compared n-tailfit, phasor analysis and n-exponential reconvolution fitting by analyzing two representative cells for n-tailfit and >20 cells for phasor and n-exponential reconvolution. A tailfit analysis may be suitable for our system because the lifetimes observed were substantially longer than the instrument response function (IRF). A setback to this method is that the tail of the decay curve may lead to inconsistencies when comparing samples across different experimental runs. We observed robust differences in the resulting lifetime for both conditions (Supplementary Table 3). Next, phasor analysis was explored (Supplementary Note 2). This approach allows for a fit free analysis of collected data and generates a visual map of the decays in the cell.[25–27] We conducted the phasor analysis on two representative conditions: An untransfected cell loaded with Cbl-4xGly-ATTO590 and a cell that was additionally transfected with a Riboglow reporter plasmid, resulting in ACTB-Ribo(4A)-590. The phasor analysis yielded robust visualization of different fluorescence lifetimes (Supplementary Table 3).

Finally, n-exponential reconvolution fit was performed. An aspect of n-exponential reconvolution fitting that was important to consider was the number of exponentials. We

assessed the quality of the fit by evaluating (i) the overlay of the fitted curve over the decay curve, (ii) a random distribution of residuals, and (iii) the lowest number of parameters. Based on this, we found a tri-exponential reconvolution fit for cells that did not contain tagged RNA and a bi-exponential reconvolution fit for cells that did contain tagged RNA to be the most appropriate (Figure 2, Supplementary Figure 5). The decision of how many parameters to use for data fitting is further influenced by considering underlying mechanistic processes that assign a meaning to the fit. In our system, both free Cbl-4xGly-ATTO590 and RNA-bound Cbl-4xGly-ATTO590 species exist in cells and binding of the RNA to the Cbl-fluorophore probe changes fluorescence intensity and fluorescence lifetime, likely due to changes in fluorescence quenching caused by fluorescence resonance energy transfer (FRET). If the interaction between the donor and acceptor is considered to be fixed, there will be only two lifetime contributing species in the system: (i) free Cbl-4xGly-ATTO590 or (ii) RNA-bound Cbl-4xGly-ATTO590, and the system is best assumed to be biexponential[28]. This further supports our decision to fit cells containing RNA to n=2 parameters. All respective data was extracted and fitted using Symphotime64 reconvolution global analysis (Picoquant).

Fitting through the n-exponential fitting yields quantitative information for further processing and analysis (Supplementary Figure 5) including amplitudes, intensities and lifetime components that can be weighted to generate the amplitude-weighted or the intensity-weighted lifetime. The resulting amplitude-weighted average lifetime was assigned to a pseudo color scale for visualization (i.e., Figure 2).

Supplementary Note 2

Phasor Analysis and fitting of FLIM-Riboglow data

Phasor analysis of fluorescence lifetime imaging (Phasor-FLIM) from time-domain measurements (method used in Picoquant Timeharp 260 acquisition card) involves Fourier transformation of the fluorescence lifetime decay curves *I*(*t*) from each pixel of the image. The phasor coordinates (**G**, **S**) are calculated using the following transformation[29].

$$\mathbf{G} = \frac{\int_0^T I(t)\cos(n\omega t)\,dt}{\int_0^T I(t)\,dt} \quad (1)$$

$$\mathbf{S} = \frac{\int_0^T I(t)\sin(n\omega t)dt}{\int_0^T I(t)dt} \quad (2)$$

where *n* is the harmonic number and represents the cycles in the laser repetition period, and ω (=2π/*T*) the angular light modulation frequency of the excitation, *T* is the period of the laser pulses (40MHz here), and *I*(*t*) is the intensity decay measured from time domain measurements (Supplementary Figure 19).

In phasor representation, the areas of the image with similar fluorescence lifetime decays (Supplementary Figure 19a) gets transferred to the similar coordinates of the phasor plot (Supplementary Figure 19b). The longer lifetimes are represented by increasing phase angle in the phasor plot (Supplementary Figure 19b). Reciprocity principle in phasor space defines that if any pixel has contribution from two dyes then the phasor position of that pixel lies along the line connecting the two phasor positions of the two dyes in the mixture. The distance from each end point is related to the inverse of the fractional intensity contribution of that dye toward the total fluorescence from that pixel (shown by the pink distribution of phasor points in Supplementary Figure 19b). If the intensity decay can be described by a single exponential (Supplementary Figure 19c), then the phasor position of that decay is on top of the universal semi-circle (Supplementary Figure 19d). Multi-exponential decays appear inside the universal semicircle.

Here, the time resolved fluorescence intensity decays obtained using the Picoquant Timeharp 260 card was converted to the phasor plot using SimFCS software (www.lfd.uci.edu).[25,27,30,31] The frequency of the excitation laser in the Abberrior

microscope is 40 MHz and the time difference between the two consecutive pulses are 25 ns. This results in a complete decay of the fluorescence intensity for the lifetimes calculated in this work (τ_{Av} = 1.5-2.5 ns) and a proper conversion to the phasor space.[32] A solution of ATTO-590 in water (τ=3.7 ns) (Sigma-Aldrich) was used for the calibration of the phasor plot and the corresponding phase and modulation corrections were applied to each of the fluorescence lifetime decays obtained in this work.[33]

To see the overall changes as an average for each cell, the average **G** and **S** coordinates of the phasor plot for each image was calculated based on the analysis described in section 4B of reference[25]. These average phasor positions are representative of the center of the heatmap of the phasor plot calculated from each of the image. These central phasor positions and their average with standard deviations are shown in Supplementary Figure 9. The data shows a shift to the longer lifetime and higher phase angle in the phasor plot after in the presence and absence of different tags.

References:

(1) Braselmann, E.; Wierzba, A. J.; Polaski, J. T.; Chromiński, M.; Holmes, Z. E.; Hung, S.-T.; Batan, D.; Wheeler, J. R.; Parker, R.; Jimenez, R.; Gryko, D.; Batey, R. T.; Palmer, A. E. A Multicolor Riboswitch-Based Platform for Imaging of RNA in Live Mammalian Cells. *Nature Chemical Biology* **2018**, *14* (10), 964–971. https://doi.org/10.1038/s41589-018-0103-7.

(2) Nakahata, Y.; Nabekura, J.; Murakoshi, H. Dual Observation of the ATP-Evoked Small GTPase Activation and Ca2+ Transient in Astrocytes Using a Dark Red Fluorescent Protein. *Scientific Reports* **2016**, *6* (1), 39564. https://doi.org/10.1038/srep39564.

(3) Poland, S. P.; Krstajić, N.; Monypenny, J.; Coelho, S.; Tyndall, D.; Walker, R. J.; Devauges, V.; Richardson, J.; Dutton, N.; Barber, P.; Li, D. D.-U.; Suhling, K.; Ng, T.; Henderson, R. K.; Ameer-Beg, S. M. A High Speed Multifocal Multiphoton Fluorescence Lifetime Imaging Microscope for Live-Cell FRET Imaging. *Biomed. Opt. Express* **2015**, *6* (2), 277–296. https://doi.org/10.1364/BOE.6.000277.

(4) Seefeldt, B.; Kasper, R.; Seidel, T.; Tinnefeld, P.; Dietz, K.-J.; Heilemann, M.; Sauer, M. Fluorescent Proteins for Single-Molecule Fluorescence Applications. *Journal of Biophotonics* **2008**, *1* (1), 74–82. https://doi.org/10.1002/jbio.200710024.

(5) Itoh, H.; Arai, S.; Sudhaharan, T.; Lee, S.-C.; Chang, Y.-T.; Ishiwata, S.; Suzuki, M.; Lane, E. B. Direct Organelle Thermometry with Fluorescence Lifetime Imaging Microscopy in Single Myotubes. *Chem. Commun.* **2016**, *52* (24), 4458–4461. https://doi.org/10.1039/C5CC09943A.

(6) Sunbul, M.; Lackner, J.; Martin, A.; Englert, D.; Hacene, B.; Grün, F.; Nienhaus, K.; Nienhaus, G. U.; Jäschke, A. Super-Resolution RNA Imaging Using a Rhodamine-Binding Aptamer with Fast Exchange Kinetics. *Nature Biotechnology* **2021**, *39* (6), 686–690. https://doi.org/10.1038/s41587-020-00794-3.

(7) Bühler, B.; Benderoth, A.; Englert, D.; Grün, F.; Schokolowski, J.; Jäschke, A.; Sunbul, M. Bright, Fluorogenic and Photostable Avidity Probes for RNA Imaging. *bioRxiv* **2021**.

(8) Paige, J. S.; Wu, K. Y.; Jaffrey, S. R. RNA Mimics of Green Fluorescent Protein. *Science* **2011**, *333* (6042), 642—646. https://doi.org/10.1126/science.1207339.

(9) Filonov, G. S.; Moon, J. D.; Svensen, N.; Jaffrey, S. R. Broccoli: Rapid Selection of an RNA Mimic of Green Fluorescent Protein by Fluorescence-Based Selection and Directed Evolution. *J. Am. Chem. Soc.* **2014**, *136* (46), 16299–16308. https://doi.org/10.1021/ja508478x.

(10) Li, X.; Kim, H.; Litke, J. L.; Wu, J.; Jaffrey, S. R. Fluorophore-Promoted RNA Folding and Photostability Enables Imaging of Single Broccoli-Tagged MRNAs in Live Mammalian Cells. *Angewandte Chemie International Edition* **2020**, *59* (11), 4511–4518. https://doi.org/10.1002/anie.201914576.

(11) Dey, S. K.; Filonov, G. S.; Olarerin-George, A. O.; Jackson, B. T.; Finley, L. W. S.; Jaffrey, S. R. Repurposing an Adenine Riboswitch into a Fluorogenic Imaging and Sensing Tag. *Nature Chemical Biology* **2022**, *18* (2), 180–190. https://doi.org/10.1038/s41589-021-00925-0.

(12) Bertrand, E.; Chartrand, P.; Schaefer, M.; Shenoy, S. M.; Singer, R. H.; Long, R. M. Localization of ASH1 MRNA Particles in Living Yeast. *Molecular Cell* **1998**, *2* (4), 437–445. https://doi.org/10.1016/S1097-2765(00)80143-4.
(13) Wu, B.; Chao, J. A.; Singer, R. H. Fluorescence Fluctuation Spectroscopy Enables Quantitative Imaging of Single MRNAs in Living Cells. *Biophysical Journal* **2012**, *102* (12), 2936–2944. https://doi.org/10.1016/j.bpj.2012.05.017.
(14) Dolgosheina, E. V.; Jeng, S. C. Y.; Panchapakesan, S. S. S.; Cojocaru, R.; Chen, P. S. K.; Wilson, P. D.; Hawkins, N.; Wiggins, P. A.; Unrau, P. J. RNA Mango Aptamer-Fluorophore: A Bright, High-Affinity Complex for RNA Labeling and Tracking. *ACS Chemical Biology* **2014**, *9* (10), 2412–2420. https://doi.org/10.1021/cb500499x.
(15) Autour, A.; C Y Jeng, S.; D Cawte, A.; Abdolahzadeh, A.; Galli, A.; Panchapakesan, S. S. S.; Rueda, D.; Ryckelynck, M.; Unrau, P. J. Fluorogenic RNA Mango Aptamers for Imaging Small Non-Coding RNAs in Mammalian Cells. *Nature Communications* **2018**, *9* (1), 656. https://doi.org/10.1038/s41467-018-02993-8.
(16) Kong, K. Y.; Jeng, S. C.; Rayyan, B.; Unrau, P. J. RNA Peach and Mango: Orthogonal Two-Color Fluorogenic Aptamers Distinguish Nearly Identical Ligands. *RNA* **2021**, *27* (5), 604–615.
(17) Chen, X.; Zhang, D.; Su, N.; Bao, B.; Xie, X.; Zuo, F.; Yang, L.; Wang, H.; Jiang, L.; Lin, Q.; Fang, M.; Li, N.; Hua, X.; Chen, Z.; Bao, C.; Xu, J.; Du, W.; Zhang, L.; Zhao, Y.; Zhu, L.; Loscalzo, J.; Yang, Y. Visualizing RNA Dynamics in Live Cells with Bright and Stable Fluorescent RNAs. *Nature Biotechnology* **2019**, *37* (11), 1287–1293. https://doi.org/10.1038/s41587-019-0249-1.
(18) Bouhedda, F.; Fam, K. T.; Collot, M.; Autour, A.; Marzi, S.; Klymchenko, A.; Ryckelynck, M. A Dimerization-Based Fluorogenic Dye-Aptamer Module for RNA Imaging in Live Cells. *Nature Chemical Biology* **2020**, *16* (1), 69–76. https://doi.org/10.1038/s41589-019-0381-8.
(19) Sunbul, M.; Jäschke, A. Contact-Mediated Quenching for RNA Imaging in Bacteria with a Fluorophore-Binding Aptamer. *Angewandte Chemie (International ed. in English)* **2013**, *52*. https://doi.org/10.1002/anie.201306622.
(20) Arora, A.; Sunbul, M.; Jäschke, A. Dual-Colour Imaging of RNAs Using Quencher- and Fluorophore-Binding Aptamers. *Nucleic Acids Research* **2015**, *43* (21), e144–e144. https://doi.org/10.1093/nar/gkv718.
(21) Sunbul, M.; Jäschke, A. SRB-2: A Promiscuous Rainbow Aptamer for Live-Cell RNA Imaging. *Nucleic Acids Research* **2018**, *46* (18), e110–e110. https://doi.org/10.1093/nar/gky543.
(22) Braselmann, E.; Palmer, A. E. A Multicolor Riboswitch-Based Platform for Imaging of RNA in Live Mammalian Cells. *Methods Enzymol* **2020**, *641*, 343–372. https://doi.org/10.1016/bs.mie.2020.03.004.
(23) Wahl, M. Time-Correlated Single Photon Counting; 2009.
(24) Wahl, M.; Orthaus-Müller, S. Time Tagged Time-Resolved Fluorescence Data Collection in Life Sciences.
(25) Ranjit, S.; Malacrida, L.; Jameson, D. M.; Gratton, E. Fit-Free Analysis of Fluorescence Lifetime Imaging Data Using the Phasor Approach. *Nature Protocols* **2018**, *13* (9), 1979–2004. https://doi.org/10.1038/s41596-018-0026-5.
(26) Hinde, E.; Digman, M. A.; Welch, C.; Hahn, K. M.; Gratton, E. Biosensor Förster Resonance Energy Transfer Detection by the Phasor Approach to Fluorescence

Lifetime Imaging Microscopy. *Microscopy Research and Technique* **2012**, *75* (3), 271–281. https://doi.org/10.1002/jemt.21054.
(27) Malacrida, L.; Ranjit, S.; Jameson, D.; Gratton, E. The Phasor Plot: A Universal Circle to Advance Fluorescence Lifetime Analysis and Interpretation. *Annual Review of Biophysics* **2021**, *50*, 575–593. https://doi.org/10.1146/annurev-biophys-062920-063631.
(28) Sillen, A.; Engelborghs, Y. The Correct Use of “Average” Fluorescence Parameters. *Photochemistry and Photobiology* **1998**, *67* (5), 475–486. https://doi.org/10.1111/j.1751-1097.1998.tb09082.x.
(29) Torrado, B.; Malacrida, L.; Ranjit, S. Linear Combination Properties of the Phasor Space in Fluorescence Imaging. *Sensors (Basel)* **2022**, *22* (3), 999. https://doi.org/10.3390/s22030999.
(30) Digman, M. A.; Caiolfa, V. R.; Zamai, M.; Gratton, E. The Phasor Approach to Fluorescence Lifetime Imaging Analysis. *Biophysical journal* **2008**, *94* (2), L14–L16.
(31) Scipioni, L.; Rossetta, A.; Tedeschi, G.; Gratton, E. Phasor S-FLIM: A New Paradigm for Fast and Robust Spectral Fluorescence Lifetime Imaging. *Nature Methods* **2021**, *18* (5), 542–550. https://doi.org/10.1038/s41592-021-01108-4.
(32) Ranjit, S.; Malacrida, L.; Gratton, E. Differences between FLIM Phasor Analyses for Data Collected with the Becker and Hickl SPC830 Card and with the FLIMbox Card. *Microscopy research and technique* **2018**, *81* (9), 980–989.
(33) Gust, A.; Zander, A.; Gietl, A.; Holzmeister, P.; Schulz, S.; Lalkens, B.; Tinnefeld, P.; Grohmann, D. A Starting Point for Fluorescence-Based Single-Molecule Measurements in Biomolecular Research. *Molecules* **2014**, *19* (10), 15824–15865. https://doi.org/10.3390/molecules191015824.
