# Supplementary material for: Visualizing orthogonal RNAs simultaneously in live mammalian cells by fluorescence lifetime imaging microscopy (FLIM)
Source: Nat Commun. 2023 Feb 16;14:867. doi: 10.1038/s41467-023-36531-y (PMC9935525; doi:10.1038/s41467-023-36531-y)
Supplement: Supplementary file 3 — Description of Additional Supplementary Files [file 41467_2023_36531_MOESM3_ESM.pdf]

Description of Additional Supplementary Files

File Name: Supplementary Data 1
Description: Sequences of NORAD variants used in this study.

File Name: Supplementary Data 2
Description: Summary of Riboglow FISH probes.
