# Supplementary material for: Visualizing orthogonal RNAs simultaneously in live mammalian cells by fluorescence lifetime imaging microscopy (FLIM)
Source: Nat Commun. 2023 Feb 16;14:867. doi: 10.1038/s41467-023-36531-y (PMC9935525; doi:10.1038/s41467-023-36531-y)
Supplement: Supplementary file 6 — Reporting Summary [file 41467_2023_36531_MOESM6_ESM.pdf]

nature portfolio

Corresponding author(s): Esther Braselmann

Last updated by author(s): Feb 3, 2023

Reporting Summary

Nature Portfolio wishes to improve the reproducibility of the work that we publish. This form provides structure for consistency and transparency in reporting. For further information on Nature Portfolio policies, see our Editorial Policies and the Editorial Policy Checklist.

Statistics

For all statistical analyses, confirm that the following items are present in the figure legend, table legend, main text, or Methods section.

n/a	Confirmed	
☐	☒	The exact sample size (*n*) for each experimental group/condition, given as a discrete number and unit of measurement
☐	☒	A statement on whether measurements were taken from distinct samples or whether the same sample was measured repeatedly
☐	☒	The statistical test(s) used AND whether they are one- or two-sided<br>*Only common tests should be described solely by name; describe more complex techniques in the Methods section.*
☒	☐	A description of all covariates tested
☒	☐	A description of any assumptions or corrections, such as tests of normality and adjustment for multiple comparisons
☐	☒	A full description of the statistical parameters including central tendency (e.g. means) or other basic estimates (e.g. regression coefficient) AND variation (e.g. standard deviation) or associated estimates of uncertainty (e.g. confidence intervals)
☐	☒	For null hypothesis testing, the test statistic (e.g. *F*, *t*, *r*) with confidence intervals, effect sizes, degrees of freedom and *P* value noted<br>*Give P values as exact values whenever suitable.*
☒	☐	For Bayesian analysis, information on the choice of priors and Markov chain Monte Carlo settings
☒	☐	For hierarchical and complex designs, identification of the appropriate level for tests and full reporting of outcomes
☒	☐	Estimates of effect sizes (e.g. Cohen's *d*, Pearson's *r*), indicating how they were calculated

Our web collection on statistics for biologists contains articles on many of the points above.

Software and code

Policy information about availability of computer code

Data collection	Microscopy images acquired on a Abberior STEDYCON instrument with Picoquant lifetime software.
Data analysis	Image analysis: ImageJ2 version 1.53 with Fiji plugin suite, Prism version 9.4.1 (458), Canvas X Draw version 2, Zotero version 6.0.17, Picoquant Symphotime 64 v.1-3 .

For manuscripts utilizing custom algorithms or software that are central to the research but not yet described in published literature, software must be made available to editors and reviewers. We strongly encourage code deposition in a community repository (e.g. GitHub). See the Nature Portfolio guidelines for submitting code & software for further information.

Data

Policy information about availability of data

All manuscripts must include a data availability statement. This statement should provide the following information, where applicable:

- Accession codes, unique identifiers, or web links for publicly available datasets
- A description of any restrictions on data availability
- For clinical datasets or third party data, please ensure that the statement adheres to our policy

All plasmids generated in this study will be deposited at Addgene. Plasmids used here that were obtained from previous studies are available from the source indicated in the Methods section. The fluorescent probe was previously developed and was provided from Amy Palmer at CU Boulder for this work (see Methods section). Access to primary data is available upon request and also available publicly on FigShare (https://doi.org/10.6084/m9.figshare.21445665.v1).

Human research participants

Policy information about studies involving human research participants and Sex and Gender in Research.

Reporting on sex and gender	The cell lines used (U-2 OS, HeLa, MDA-MB231) are immortalized cell lines and therefore sex and gender reporting is not applicable, per the SAGER guidelines.
Population characteristics	Not applicable.
Recruitment	Not applicable.
Ethics oversight	Not applicable.

Note that full information on the approval of the study protocol must also be provided in the manuscript.

Field-specific reporting

Please select the one below that is the best fit for your research. If you are not sure, read the appropriate sections before making your selection.

☒ Life sciences ☐ Behavioural & social sciences ☐ Ecological, evolutionary & environmental sciences

For a reference copy of the document with all sections, see nature.com/documents/nr-reporting-summary-flat.pdf

Life sciences study design

All studies must disclose on these points even when the disclosure is negative.

Sample size	A central goal of this study was systematic comparison of different data analysis approaches for the same dataset to determine which is the most robust (Supp. Table 3). Therefore, a predetermination of sample size based on expected effects of analyzed data was not possible. To estimate sample size, we compared similarly designed fluorescence lifetime imaging microscopy studies in the literature with our work and chose sample sizes larger than those reported in comparable studies to make sure we were sampling enough. We use more than 50 (up to over 100) cells for each condition. Comparable studies in the literature used roughly 10-25 cells per condition, so we were confident that our sample size is sufficient.
Data exclusions	Cells that were dying/dead were excluded. These cells were identified visually by fluorescence microscopy imaging and typically occurred at late stages of imaging sessions.
Replication	All attempts of data replication were successful, indicated in figure legends.
Randomization	We included randomization when feasible for our experiments. For example, we typically performed experiments for at least two different conditions in parallel using cells that were split from the same dish and chosen at random.
Blinding	Blinding was not feasible due to the complexity of data collection and because processing and all steps were frequently done by the same person. To validate robustness, a representative blind run was performed for a set of experimental conditions at the center of this study (figure S 10).

Reporting for specific materials, systems and methods

We require information from authors about some types of materials, experimental systems and methods used in many studies. Here, indicate whether each material, system or method listed is relevant to your study. If you are not sure if a list item applies to your research, read the appropriate section before selecting a response.

Materials & experimental systems

n/a	Involved in the study	
☒	☐	Antibodies
☐	☒	Eukaryotic cell lines
☒	☐	Palaeontology and archaeology
☒	☐	Animals and other organisms
☒	☐	Clinical data
☒	☐	Dual use research of concern

Methods

n/a	Involved in the study	
☒	☐	ChIP-seq
☒	☐	Flow cytometry
☒	☐	MRI-based neuroimaging

Eukaryotic cell lines

Policy information about cell lines and Sex and Gender in Research

Cell line source(s)	U-2 OS cells: tissue culture and biobanking shared resource, Georgetown University.<br>U-2 OS Halo-G3BP1 cells: Roy Parker, CU Boulder.<br>MDA-MB231 cells: tissue culture and biobanking shared resource, Georgetown University.<br>HeLa cells: tissue culture and biobanking shared resource, Georgetown University.
Authentication	Cell lines were authenticated by source (above). We did not use cells for longer than 12 passages.
Mycoplasma contamination	All cells are stored at liquid nitrogen and used upon storing aliquots of the frozen stocks. Mycoplasma testing was performed every time vials were thawed. All tests for mycoplasma were negative.
Commonly misidentified lines<br>(See ICLAC register)	No commonly misidentified cell lines were used in this study.
